# Total Flavonoids from *Rhizoma Drynariae* (Gusuibu) Alleviates Diabetic Osteoporosis by Activating BMP2/Smad Signaling Pathway

**DOI:** 10.2174/1386207326666230223165730

**Published:** 2023-06-20

**Authors:** Xin Hua Fang, Guo Er Zhou, Na Lin

**Affiliations:** 1 Hangzhou TCM Hospital Affiliated to Zhejiang Chinese Medical University Hangzhou, Zhejiang 310015, China;; 2 Zhoushan TCM Hospital Affiliated to Zhejiang Chinese Medical University, Zhejiang 316100, China;; 3 Lishui TCM Hospital Affiliated to Zhejiang Chinese Medical University, Zhejiang, 323000, China

**Keywords:** *Rhizoma Drynariae* (Gusuibu), Diabetic osteoporosis (DOP), BMP2/Smad signaling pathway, Bone homeostasis, histopathology, osteoporosis

## Abstract

**Introduction:**

Diabetic osteoporosis (DOP) is a widespread public health problem. The flavonoids of *Rhizoma Drynariae* (RDF) have a clear preventive and therapeutic effect on osteoporosis (OP), but it is not yet clear whether RDF has an anti-DOP and whether its mechanism is related to the activation of the BMP2/Smad signaling pathway. The current study aimed to study this effect of RDF in DOP rats and the possible involvement of the BMP2/Smad signaling pathway activation.

**Methods:**

Following intragastric administration of RDF for 12 weeks, the body weight, blood glucose, and the bone histopathological changes detected by hematoxylin-eosin (H&E) and calcein staining were monitored, while bone parameters were regularly assessed from observations made by micro-CT. At the end of the experiment, the expression of *Bmp2, Bmpr1a, Runx2,* and *Smad4/5* genes was detected by real-time PCR (RT-PCR). Meanwhile, western blotting or immunohistochemical staining monitored the protein expressions of BMP2, RUNX2, and SMAD5 in the bone.

**Results:**

The results firstly indicated that RDF significantly alleviated the signs and symptoms of DOP, which manifested as improved body weight and blood glucose. As obtained from the results of histopathology and micro-CT, RDF could promote the formation of bone trabeculae and alter several the bone microstructure parameters, including an increase in the bone volume/total volume (BV/TV), connective density (Conn-Dens), and trabecular bone number (Tb.N), as well as a decrease in the trabecular spacing (Tb.Sp). The western blotting analysis and RT-PCR results also confirmed that RDF could markedly increase the mRNA expression levels of *Bmp2*, *Bmpr1α*, *Smad4*, *Runx2*, and *Smad5* in the bone, as well as the corresponding protein expression levels of BMP2, RUNX2, and SMAD5. These results reveal that RDF can activate the BMP2/Smad signaling pathway, thus promoting bone remodeling in DOP rats.

**Conclusion:**

RDF can increase bone trabeculae and bone mineral density by promoting bone formation and inhibiting bone absorption, thereby playing a role in improving DOP. This effect is related to the regulation of the BMP2/Smad signaling pathway.

## 1 INTRODUCTION

DOP is an important complication of diabetes mellitus that is characterized by reduced bone formation, thereby rendering bones prone to fracture and seriously reducing the quality of life of affected patients [1]. In 1948, Albright first reported that osteoporosis can occur in patients experiencing poor long-term control of their diabetes poor long-term control of their diabetes [2]. The prevalence of osteoporosis in diabetes mellitus patients is reportedly 37.8%~50% [3]. At present, DOP is often treated with basic hypoglycemic drugs combined with drugs that inhibit bone absorption. Still, this strategy further reduces bone turnover rate and an increased fracture risk, thus having obvious shortcomings [4].

Meanwhile, calcitonin, estrogen, and raloxifene are commonly used to treat osteoporosis. However, the adverse reactions caused by these drugs seriously restrict their clinical application [5]. For example, estrogen can maintain bone mineral density but increases the risk of cancer, thrombosis, and heart disease. With the increasing prevalence of diabetes, the burden of DOP is increasing in tandem worldwide and has thus received extensive attention to developing new strategies for its prevention and treatment.

The pathogenesis of DOP is complex, which makes it difficult to treat. The imbalance in bone homeostasis that is associated with diabetes is well known to be caused by more osteoclast-mediated bone resorption when compared to osteoblast-mediated bone formation results in both OP and DOP [6]. Many factors lead to bone homeostasis, but the fundamental mechanism involves an imbalance between osteoblasts (OBs) and osteoclasts, including decreased OBs differentiation and activity, thereby resulting in reduced bone deposition [6]. The bone morphogenetic protein (BMP)/ Smad family members (Smads) signalling pathway plays an important role inactivating the transcription of genes that underly bone formation [7]. Bone morphogenetic protein II (BMP2), a member of the TGF-β superfamily, is highly expressed in OB and promotes osteoblast differentiation and the synthesis and secretion of the bone extracellular matrix [8]. BMP binds to heterodimeric receptors (BMPRs), specifically binding to BMPR1 to activate SMAD proteins, which then transactivate osteoblastogenic genes either directly or via Runt-related transcription factor 2 (RUNX2) [9]. RUNX2 is the downstream target gene of the BMP2/Smad signal transduction, and it is a key and transcription factor involved in the differentiation of bone marrow mesenchymal stem cells (BMSCs) into osteoblasts. RUNX2 can upregulate the transcription of various genes encoding mineralization-related proteins in chondrocytes and pre-osteoblasts to allow them to differentiate into osteoblasts [10]. Clinical and animal studies have shown that activating the BMP2/Smad signaling pathway effectively controls DOP [11].

Traditional Chinese medicine (TCM) has emerged as an active area of research in the context of OP due to commonly resulting in a good overall treatment effect, low rate of adverse reactions, and suitability for long-term use [12]. *Drynaria fortunei (Kunze)* J.Sm*.,* also known as Gusuibu, is a classic TCM with a long medical history used in orthopedics and traumatology. *Drynaria fortunei (Kunze)* J. Sm. is obtained from the dry rhizoma of *Drynaria fortune*, which belongs to the polypodiaceae family. The herb contains flavonoids, lignans, steroids, phenylpropanoids, triterpenes, and other main active ingredients, and the flavonoids of *Rhizoma Drynariae* (RDF) are the main active ingredients of anti-OP. In OP model rats, RDF has been reported to reduce the apoptosis of osteoblasts induced by retinoic acid effectively, inhibit the serum cathepsin K concentration, and increase the level of BMP [13, 14]. More significantly, several clinical studies have found that RDF could improve bone mineral density to exert a therapeutic effect on osteoporotic fractures [15]. Thus, it can be seen that the RDF has a clear preventive and therapeutic effect on OP. However, it remains unclear whether RDF also has an anti-DOP effect and whether the underlying mechanism of this effect is related to the activation of the BMP/Smad signaling pathway.

Therefore, this study first established a DOP rat model to investigate these questions. Measurements were taken of body weight, blood glucose, and the pathological changes of DOP induced by RDF treatment. At the same time, analyses were also conducted using H&E staining, calcein staining, and investigating changes in bone parameters by using micro-CT, as well as the detection of the gene and protein expression levels of BMP2, BMPR-1a, RUNX2, and SMAD4/5 by both RT-PCR and Western blotting (WB). The data were used to elucidate the mechanism of RDF in preventing and treating DOP by activating the BMP/Smad signaling pathway. The findings of this study reveal the underlying mechanism and support the clinical application of RDF in preventing and treating DOP.

## 2 MATERIALS AND METHODS

### 2.1 Establishment and Grouping of the DOP Model

A total of 30 male specific-pathogen-free SD rats were purchased from Shanghai Shrek Laboratory Animal Co., Ltd. The rats used in the present study were treated in accordance with the Guide for the Care and Use of Laboratory Animals. All animal experiments were performed with the approval of the Bioethics Committee of the Zhejiang Academy of Traditional Chinese Medicine (Approval No: KTSB2021040) in the 2021 year.

After 30 days of adaptive feeding, 24 rats were fed for 4 weeks with a high-sugar and high-fat diet (Cat#1135D, Boaigang, Beijing, China). A 0.1 mol/L sodium citrate solution prepared a 1% Streptozotocin (STZ, Cat#S0130, Boaigang) solution. Fresh 1% STZ solution was then injected intraperitoneally (ip) at 35 mg/kg to induce a diabetic rat model reference to Hao [16]. The modeled rats were continuously fed with a high-sugar and high-fat diet, while another 6 rats were fed with normal standard feeding.

Then blood glucose was measured from the tail vein on days 3, 5, and 7. The diabetes mellitus model was determined to be successfully established when three successively obtained blood glucose measurements all exceeded 11.0 mmol/L. The rats were divided into three groups, which had an even mixture of weights and blood glucose levels, following as low-dose RDF treatment group (RDF-L group, n = 6), high-dose RDF treatment group (RDF-H group, n = 6), and DOP model group (MG, n = 6). The RDF-H and RDF-L groups of rats were intraperitoneally administrated 100 and 200 mg/kg/day daily for 12 weeks, respectively. Body weights were measured every week and blood glucose was measured from the tail vein every two weeks over the course of the treatment.

RDF was purchased from Xian Kailai Bio-Technology Co., Ltd. Purity was approximately 99.50%. The MG has administrated with normal saline under the same conditions.

### 2.2 Micro-CT Scanning

At the end of the experiment, the right femur was quickly removed and fixed in a 4% paraformaldehyde solution. Subsequently, the trabecular microstructure of the tibia was scanned using an *in vitro* micro-CT tomography technique (SkyScan 1176, Institute of Hydrobiology, Chinese Academy of Sciences). The voltage of the scanner was 70 Kv, the scanning current was 200 *μ*A, the layer spacing was 18.0 *μ*m, and the planar resolution was 300 ms. CT analysis using Airborne software was conducted to detect trabecular microstructure parameters: BV/TV, Tb.N, and Tb.Sp.

### 2.3 Calcein Staining

On the 10th weeks after the ration of RDF, three animals per group received 20 mg/kg of calceinbyintra (Cat#108750-13-6, Sangon, Shanghai, China) solution muscular injection. At the end of the experiment, the rats' right femurs were quickly removed and scanned by the Molecular Devices ImageXpress^®^ Pico system (Molecular Devices, USA).

### 2.4 Histomorphometric Evaluation

After the rats were sacrificed, their femurs were obtained and fixed in 4% formalin for 48 hours before being immersed in 10% EDTA for 30 days. Then, the bone tissues were embedded and the paraffin blocks were cut into 4 μm sections. The resulting paraffin sections then underwent H&E staining (Cat#R20570, Yuanye, Shanghai, China) before being observed under an inverted microscope (Olympus, Tokyo, Japan).

### 2.5 Real-Time PCR

In brief, the total RNA of bone was extracted using an RNA Purification Kit (Cat#RN54, aidelai, Beijing, China) and the SPARK Script II Strand cDNA Synthesis Kit (Cat#AC0205-B, Sikejie, Shangdong, China) was used to obtain cDNA. Subsequently, the cDNA was amplified using SYBR Green qPCR Master Mix (Cat#AH0104B, Sikejie, Shangdong, China) on an RT-PCR Detection System (ABI system, Waltham, USA) in a 10 μL reaction system. The PCR conditions were set according to the manufacturer's instructions. β-actin was used as a normalization control and the fold change for each target gene was calculated using the 2^−ΔΔCt^ method. The sequences of the mRNA primers are displayed in Table **1**.

### 2.6 Western Blotting

Left tibia was crushed and the total protein was extracted with a special bone tissue protein extraction kit (Cat#HRO117, Baiao Leibo, Beijing, China,). A BCA protein assay kit (Cat#KGBCA, KeyGEN, Jiangsu, China) was used to quantify the total protein. The approximately 30 μg of the total protein was subjected to SDS-PAGE, and the resulting protein bands were immunoblotted with primary antibodies against GAPDH (Cat#AF0006, Beyotime, Shanghai, China, 1:1000 dilution), BMP2 (Cat#GB11252, Servicebio, Hubei, China, 1:1000 dilution,),SMAD5 (Cat#AF1405, Beyotime, Shanghai, China, 1:1000 dilution) and RUNX2 (Cat#AF2593, Beyotime, Shanghai, China, 1:1000 dilution) at 4°C overnight. The membranes were then washed with anti-rabbit-HRP conjugated secondary antibodies (Cat#A0208, Beyotime, Shanghai, China, 1:2000 dilution), or Anti-mouset-HRP conjugated secondary antibodies (Cat#A0216, Beyotime, Shanghai, China, 1:2000 dilution). Band images were detected by the ChemiDoc™ MP Imaging system (Bio-Rad). The gray values of the protein band were obtained by the software image J.

### 2.7 Immunohistochemical Staining

The expression of BMP2, BMPR1a and RUNX2 inbone was determined by immunohistochemical staining, as others described [17]. The deparaffinized tissue sections were incubated with BMP2 rabbit primary antibody (Cat#GB11252, Servicebio, Hubei, China), BMPR1a rabbit primary antibody (Cat#R1510-1, HUABIO, Zhejiang, China), and RUNX2 rabbit primary antibody (Cat#AF2593, Beyotime, Shanghai, China). Secondary HRP-conjugated goat anti-rabbit IgG (Cat#SV0002, BOSTER, Beijing, China) was then added. The signals were visualized by DAB staining(Cat#SV0002, BOSTER, Beijing, China) and the nuclei were counterstained with hematoxylin. Positive staining presented with a yellow color under a microscope(Olympus, Tokyo, Japan).

### 2.8 Statistical Analysis

All data were analyzed using SPSS 19.0 (SPSS Inc., Chicago, IL, USA) and are expressed as the mean±s.d. Statistical significance was the Student’s t-test or one-way analysis of variance (ANOVA). Statistical significance was defined as *P*<0.05.

## 3 RESULTS

### 3.1 RDF Alleviated DOP

**Fig. (1):**
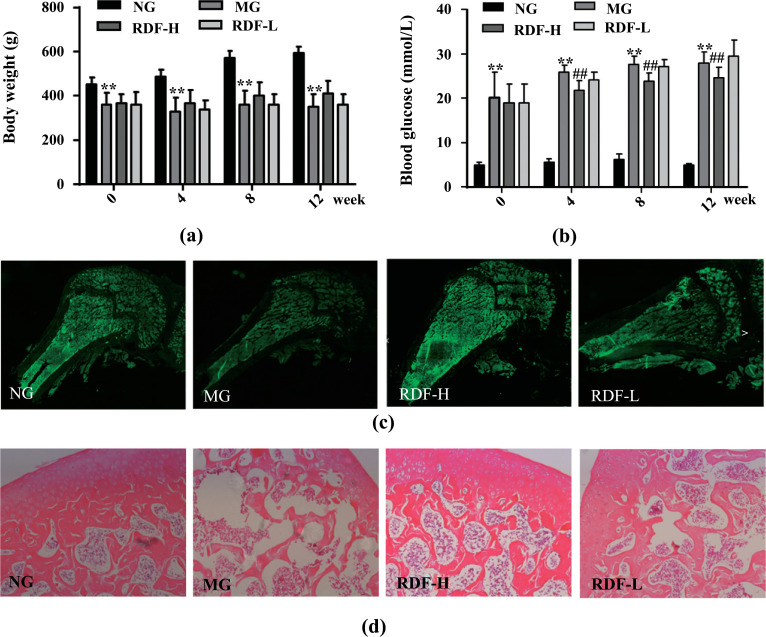
Effect of RDF in DOP Rats. (**a**) Change in body weight. (**b**) Change in blood glucose. (**c**) Calcein staining (400×). (**d**) H&E staining (200×). ^∗^*P* < 0.05 and ^∗∗^*P* < 0.01 compared to the normal group (NG); ^#^*P* < 0.05 and ^##^*P* < 0.01 compared to the model group (MG).

In this study, we measured the body weights and blood glucose levels of rats treated with RDF. Compared to normal rats, body weight and blood glucose were significantly lower in the DOP model rats, while RDF reversed these outcomes (Fig. **1a** and **b**). H&E staining (Fig. **1d**) showed abundant, continuous, and dense trabecular bone in the NG rats, while the DOP group instead showed a larger marrow cavity and a decreased number of trabecular bones, which are typical characteristics of osteoporosis. Compared to MG rats, RDF treatment for 12 weeks reversed the features of osteoporosis. To further confirm the presence of osteoporosis, calcein staining was performed (Fig. **1c**), from which similar results were obtained to show that RDF treatment improved osteoporosis. These results further indicate that RDF can alleviate DOP.

### 3.2 RDF Improved Trabecular Bone Parameters in DOP Rats

**Fig. (2):**
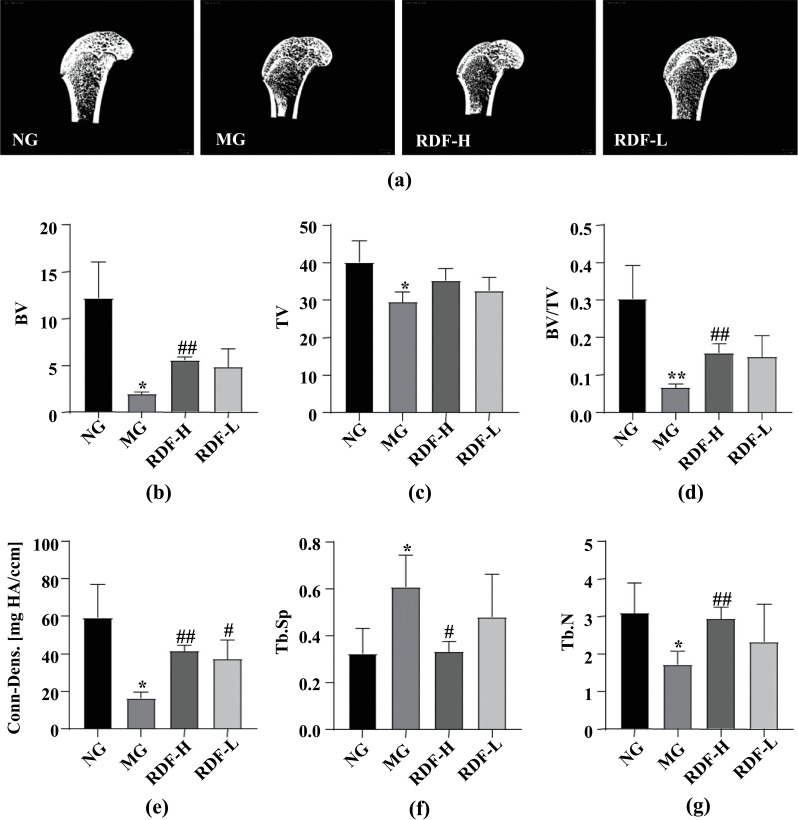
Effect of RDF on trabecular bone parameters in the DOP Rats. (**a**) Representative pictures of Micro-CT test trabecular bone parameters in rats. (**b**) Bone volume (BV). (**c**) Tissue volume (TV). (**d**) Bone volume/total volume (BV/TV). (**e**) Trabecular connection density (Conn-Dens). (**f**) Degree of trabecular separation (Tb.Sp). (**g**) Trabecular number (Tb.N). ^∗^*P*< 0.05 and^∗∗^*P* < 0.01, compared with the normal group (NG); ^#^*P*< 0.05 and ^##^*P*< 0.01, compared with the model group (MG).

Trabecular bone parameters were analyzed by micro-CT (Fig. **2**), and the results showed that the DOP group had significantly lower values for BV, TV, BV/TV, connective density (Conn-Dens), and Tb.N, while higher Tb.Sp was observed in the MG (*P* < 0.05, 0.01) compared to the NG. Compared to the MG, the RDF-H group exhibited a significant reversal in the changes in the trabecular bone parameters of BV, BV/TV, Conn-Dens, Tb.N, and Tb.Sp (*P* < 0.05, 0.01). The RDF-L group similarly exhibited significantly increased Conn-Dens compared to the MG (*P* < 0.05). These results show that RDF promoted the formation of bone trabeculae and increased the bone microstructure parameters BV/TV, Conn-Dens, and Tb.N, while decreasing that of Tb.Sp in rats. This finding suggested that RDF improves DOP by promoting bone formation and inhibiting bone absorption.

### 3.3 RDF Alleviated Changes in BMP2/Smad mRNA Expression in DOP Rats

**Fig. (3):**
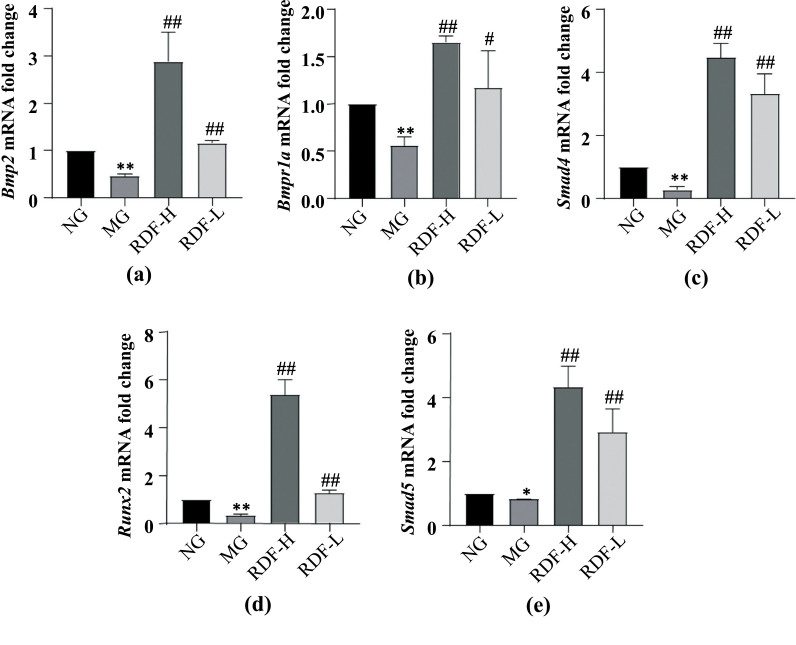
Effect of RDF on the bone of BMP2/BMPR1a mRNA in the DOP Rats. (**a**) The level of bone *Bmp2* mRNA. (**b**) The level of bone *Bmpr1a* mRNA. (**c**) The level of bone *Smad4* mRNA. (**d**) The level of bone *Runx2* mRNA. (**e**) The level of bone *Smad5*mRNA. ^∗^*P*< 0.05 and^∗∗^*P* < 0.01, compared to the normal group (NG); ^#^*P*< 0.05 and ^##^*P*< 0.01, compared to the model group (MG).

We also examined the mRNA expression levels of the BMP/Smad signaling pathway in DOP rats. The results showed that, when compared to the NG, the MG exhibited significantly decreased mRNA expression levels of *Bmp2, Bmpr1α, Smad4, Runx2,* and *Smad5* in the bone (*P* < 0.05, 0.01) (Fig. **3**). Compared to the MG, the RDF group had significantly increased mRNA expression levels in the bone in a dose-dependent manner (*P* < 0.05, 0.01) (Fig. **3**). These results suggest that RDF activates the BMP2/Smad signaling pathway, thus promoting the recovery of DOP.

### 3.4 RDF Alleviated Expression Changes of BMP2/Smad Proteins in DOP Rats

**Fig. (4):**
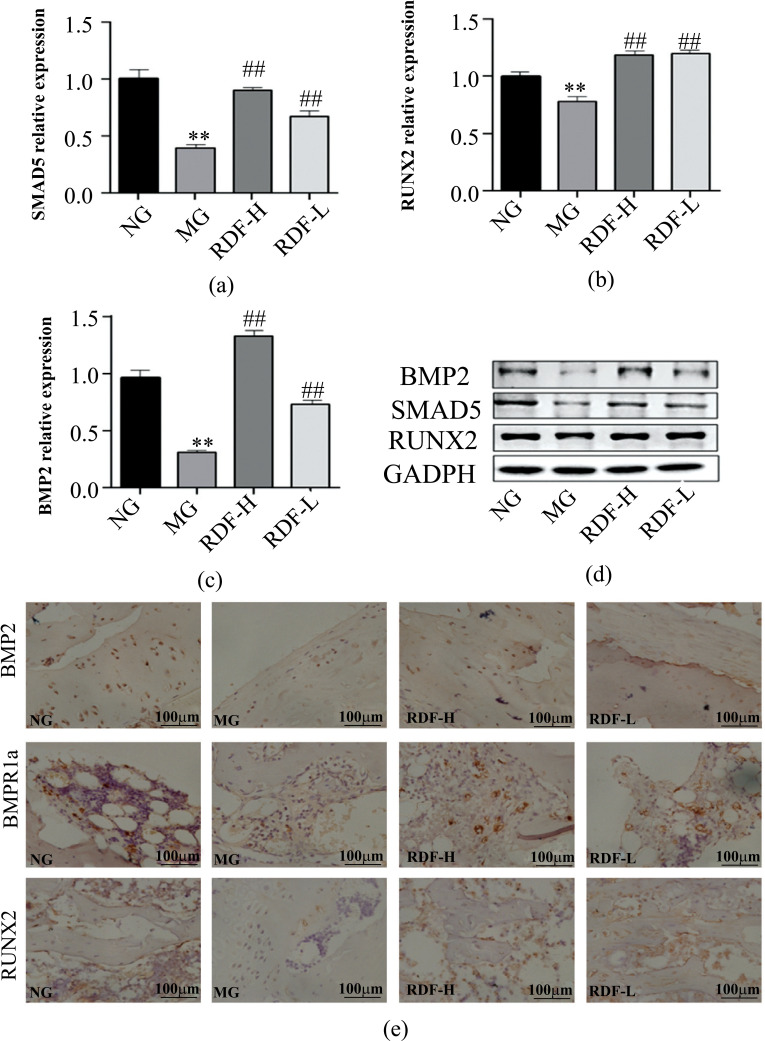
Effect of RDF on the bone of BMP2, RUNX2, and SMAD5 protein in the DOP Rats. (**a**) The level of bone SMAD5. (**b**) The level of bone RUNX2. (**c**) The level of bone BMP2. (**d**) The representative strip of WB of bone BMP2, RUNX2, and SMAD5. (**e**) The representative expression of BMP2, BMPR1α, and RUNX2 of IHC (400×). ^∗^*P*< 0.05 and ^∗∗^*P* < 0.01 compared to the normal group (NG); ^#^*P* < 0.05 and ^##^*P* < 0.01 compared to the model group (MG).

We also examined the protein expression levels of BMP2, RUNX2, and SMAD5 in the BMP/Smad signaling pathway in DOP rats. Compared to the NG, the MG exhibited significantly decreased expression levels of BMP2, RUNX2, and SMAD5 in the bone (*P* < 0.01) (Fig. **4**). Compared to the MG, the RDF group significantly increased their expression levels dose-dependently in the bone (*P* < 0.01) (Fig. **4**). The IHC results found that the expression levels of BMP2, BMPR1α, and RUNX2 were lower in the bone of the MG, while RDF-H and RDF-L consumption reversed these parameter changes. These results suggest that RDF activates the BMP2/Smad signaling pathway, thus promoting the recovery of DOP.

## 4 DISCUSSION

Diabetic osteoporosis (DOP) is a worldwide public health problem with high prevalence [1]. The clinical manifestations of DOP include bone absorption exceeding bone formation, bone formation decreasing, and bone turnover being downregulated. The mechanism by which osteoporosis is caused by diabetes is complex. For example, long-term hyperglycemia can lead to calcium, phosphorus, and magnesium loss, thereby leading to bone loss and osteoporosis. Osteoblasts are the main functional cells involved in bone formation, while BMSCs are important precursor cells to osteoblasts. Inhibition of the osteogenic differentiation of BMSCs to osteoblasts induced by high glucose, oxygen free radicals, and inflammatory factors may be important factors that contribute to DOP. For example, a high glucose environment can significantly inhibit the growth of BMSCs in a dose-dependent manner [18]. Hyperglycemia leads to the excessive accumulation of advanced glycation end products, which inhibit the expression of the osteoblast phenotype and reduce bone formation [19-21]. This suggests that reducing blood glucose and improving osteoblast or BMSCs proliferation and function will be effective ways to promote bone formation in diabetes.

Increasing evidence shows that the use of Traditional Chinese medicine (TCM) is effective for treating diseases, such as in increasing osteogenic differentiation and activity in DOP. *Drynaria fortunei (Kunze)* J. Sm., also known as Gusuibu, is a classic TCM used in the treatment of bone disease. Sun *et al* reported that RDF could ameliorate bone formation and mineralization in large tibial defect rats. It has also been reported that RDF has a clear preventive and therapeutic effect on OP [22], while its effect on DOP has yet to be reported. In this study, we found that treatment with a high dose of RDF could significantly decrease the blood glucose level of rats after 4 weeks, and this hypoglycemic effect was sustained for 12 weeks. Furthermore, RDF could alleviate pathological bone changes by increasing abundant, continuous, and dense trabecular bone, thus promoting bone formation in DOP rats. These results indicate that RDF can significantly ameliorate several features of DOP, thereby serving as a novel prospective drug for DOP treatment.

The trabecular bone is an important structure in cancellous bone, and its structural integrity is vital for maintaining bone strength and reducing the incidence of fracture. The microstructure of trabecular bone is of great value for diagnosing and treating OP [23]. It has been reported that patients with diabetes have a lower trabecular bone score, while a higher trabecular bone score is negatively correlated with blood glucose. Changes in the bone microstructure parameters are of great significance for predicting changes in bone strength [24]. BV/TV is a common indicator that reflects the amount of trabecular bone in different samples. If BV/TV increases, it indicates that bone anabolism is greater than bone decomposition; thus, bone mass increases as a result. By contrast, if BV/TV decreases, bone anabolism is less than bone decomposition, and bone mass decreases as a result [25]. Conn-Dens, Tb.N, and Tb.Sp are the main indexes used to evaluate the spatial morphology of trabecular bone. In OP and DOP, the values of Conn-Dens, Tb.N, and Tb.S decrease [25]. Through analyzing the above parameters, this experiment found that intervention by RDF administration could significantly improve bone parameters related to bone microstructure, suggesting that it increases the number and thickness of bone trabeculae, reduces the separation of the bone trabeculae, and improves the orientation, symmetry, and morphological structure of bone trabeculae to enhance bone density, thus exerting its anti-DOP effect.

The BMP/Smad signalling pathway is important in activating osteogenic differentiation and bone formation. BMP2 and a variety of cytokines comprise the BMP2 signaling pathway, which can promote osteoblast differentiation, synthesis, and the secretion of the bone extracellular matrix. Clinically, OP patients exhibit decreased bone mineral density, which is accompanied by a decrease in the BMP2 level, while treatment can improve bone mineral density by increasing BMP2 [26]. The Food and Drug Administration has approved the usage of recombinant human BMP2 (rhBMP2) during tibial shaft repair [27]. BMP2 primarily acts by binding to serine/threonine receptors (BMPRs) on the surface of target cells. For example, BMP2 binds to BMPR-I and then activates phosphorylates of Smads, which are then transferred into the nucleus to upregulate RUNX2, which is a key factor necessary for the osteogenic differentiation of BMSCs and bone development.

In the present study, we evaluated the effect of RDF on the BMP/Smad signaling pathway in preventing DOP. We found that treatment with RDF markedly up-regulated the gene expression levels of *Bmp2*, *Bmpr1α*, *Smad4*, *Runx2*, and *Smad5* in DOP rats. Moreover, treatment with RDF increased the protein expression levels of BMP2, BMPR1α, SMAD5, and RUNX2. These findings indicate that RDF can promote bone formation to maintain the balance of bone metabolism by regulating the BMP2/Smad signaling pathway.

## CONCLUSION

In summary, we found that RDF could decrease the blood glucose level, improve body weight, promote the formation of bone trabeculae, and increase the bone microstructure parameters of BV/TV, Conn-Dens, and Tb.N while decreasing that of Tb.Sp. These findings suggest that RDF can increase bone trabeculae and bone mineral density by promoting bone formation and inhibiting bone absorption, thereby improving DOP. Its mechanism of action may be related to its regulation of the BMP2/Smad signaling pathway. This study, therefore, promotes the clinical application of RDF in preventing and treating DOP by revealing its underlying mechanism. However, this study is limited because it has only employed a rat model, such as. Further *in vitro* experiments should subsequently be conducted to observe the effect and regulatory mechanism of RDF on osteoblasts and BMSC osteogenic differentiation induced by high glucose.

## AUTHORS’ CONTRIBUTIONS

Na Lin conceived and designed the experiments. Xin hua Fang executed and wrote the manuscript preparation. Guo er Zhou helps to analyse the data.

## Figures and Tables

**Table 1:** Sequences of the primers of mRNA used in the study.

**Gene name**	**Gene ID**	**Forward**	**Reverse**
*Bmpr1a*	81507	GCTGTGCTCATCTCTATGGCTGTC	TCCTGTTCCAAGTCACGGTTGTAAC
*Bmp2*	29373	AACCTGCAACAGCCAACT	GCTCAGTGTAGCCCAGGAT
*Runx2*	367218	TGATGCGTATTCCCGTAGA	CATGGTGCGGTTGTCG
*Smad4*	50554	CAGCCAGGACAGCAGCAGAATG	TGGTGGTGAGGCAAATTAGGTGTG
*Smad5*	59328	GCTTCTGGCTCAGTCAGTCAACC	ATCCTGTCGGTGGTACTCTGCTC
*β-actin*	81822	CGTAAAGACCTCTATGCCAACAC	CGGACTCATCGTACTCCTGCT

## Data Availability

The data to support the findings of this study are included in the article. Other data used to support the findings of this study are available from the corresponding author upon request.

## LIST OF ABBREVIATIONS

BMP2: Bone Morphogenetic Protein II
BMPR: Binding to Serine/Threonine Receptor
BMSCs: Bone Marrow Mesenchymal Stem Cells
BV: Bone Volume
BV/TV: Bone Volume/Total Volume
Conn-Dens: Connective Density
DOP: Diabetic Osteoporosis
H&E: Hematoxylin-Eosin
OP: Osteoporosis
RDF: Flavonoids of *Rhizoma Drynariae*
RUNX2: Runt-Related Transcription Factor 2
Tb.N: Trabecular Bone Number
Tb.Sp: Trabecular Spacing
TCM: Traditional Chinese Medicine
TV: Tissue Volume
WB: Western Blotting
